# Repurposing of the anti-malaria drug chloroquine for Zika Virus treatment and prophylaxis

**DOI:** 10.1038/s41598-017-15467-6

**Published:** 2017-11-17

**Authors:** Sergey A. Shiryaev, Pinar Mesci, Antonella Pinto, Isabella Fernandes, Nicholas Sheets, Sujan Shresta, Chen Farhy, Chun-Teng Huang, Alex Y. Strongin, Alysson R. Muotri, Alexey V. Terskikh

**Affiliations:** 1 Sanford Burnham Prebys Medical Discovery Institute, 10901 N. Torrey Pines Rd., La Jolla, CA 92037 USA; 2 University of California San Diego, School of Medicine, Department of Pediatrics/Rady Children’s Hospital San Diego, Department of Cellular & Molecular Medicine, Stem Cell Program, La Jolla, CA 92037-0695 USA; 3 Division of Inflammation Biology, La Jolla Institute for Allergy & Immunology, La Jolla, CA 92037 USA

## Abstract

One of the major challenges of the current Zika virus (ZIKV) epidemic is to prevent congenital foetal abnormalities, including microcephaly, following ZIKV infection of pregnant women. Given the urgent need for ZIKV prophylaxis and treatment, repurposing of approved drugs appears to be a viable and immediate solution. We demonstrate that the common anti-malaria drug chloroquine (CQ) extends the lifespan of ZIKV-infected interferon signalling-deficient AG129 mice. However, the severity of ZIKV infection in these mice precludes the study of foetal (vertical) viral transmission. Here, we show that interferon signalling-competent SJL mice support chronic ZIKV infection. Infected dams and sires are both able to transmit ZIKV to the offspring, making this an ideal model for *in vivo* validation of compounds shown to suppress ZIKV in cell culture. Administration of CQ to ZIKV-infected pregnant SJL mice during mid-late gestation significantly attenuated vertical transmission, reducing the ZIKV load in the foetal brain more than 20-fold. Given the limited side effects of CQ, its lack of contraindications in pregnant women, and its worldwide availability and low cost, we suggest that CQ could be considered for the treatment and prophylaxis of ZIKV.

## Introduction

The recent re-emergence of Zika virus (ZIKV) represents a public health emergency^1^. ZIKV is a member of the *Flaviviridae* family of viruses, which are transmitted to humans by mosquitoes and ticks and are responsible for millions of infections annually. ZIKV has historically been restricted to tropical/sub-tropical regions but it has now reached the Southern USA^2^. Recent studies have demonstrated that ZIKV infects and predominantly damage human neural precursors; however, the cellular and molecular mechanisms of ZIKV pathogenesis remain poorly understood^3–6^.

ZIKV infection causes moderately severe disease in about 20% of infected adults and is normally accompanied by mild symptoms such as headache, low fever, malaise, skin rashes, conjunctivitis, and muscle and joint pain^7^. More alarming is the evidence linking ZIKV infection in pregnant women to severe microcephaly in the foetus. The number of birth defects in children born to ZIKV-infected mothers is about 20-fold higher than normal^8^. In addition, ZIKV infection of adults can cause neuronal pathology such as Guillain-Barré syndrome, a rapid-onset acute polyneuropathy caused by an autoimmune response to the peripheral nervous system^9,10^.

In the absence of established anti-ZIKV treatments, repurposing of available drugs approved for use in pregnant women is likely to be the most effective way to reduce ZIKV infection in adults and to limit birth defects in newborns. Chloroquine (CQ) phosphate/CQ hydrochloride (Aralen) has a long and successful history as an oral anti-malarial chemotherapy, and there are no substantiated reports that CQ used for malarial prophylaxis^11^ causes foetal harm. CQ is also prescribed for the treatment of rheumatoid arthritis and other autoimmune disorders. Recently, CQ was suggested to inhibit ZIKV infection in several cell models, including human neural precursor cells (NPCs) derived from induced pluripotent stem cells (iPSCs)^12^. CQ was also shown to inhibit autophagy and ZIKV propagation in interferon signalling-deficient pregnant mice^13^. Here, we demonstrate that administration of CQ significantly extended the lifespan of interferon (IFN) type I and II receptor-deficient AG129 mice infected with Brazilian strain ZIKV (ZIKV^BR^, Brazil-ZKV2015), a common preclinical model for ZIKV research. However, the severity of disease precludes the use of AG129 mice for the investigation of vertical ZIKV transmission. To develop a suitable model for this purpose, we used SJL mice, which have a normal IFN signalling response and have previously been used for the study of ZIKV pathogenesis^4^. We found not only that SJL mice support chronic ZIKV^BR^ infection but also that the virus can be transmitted vertically, making this a more relevant model of ZIKV infection in humans^14^. Notably, administration of CQ to pregnant SJL mice during mid-late gestation markedly reduced ZIKV^BR^ infection in the foetal brain. Collectively, our data suggest that CQ could be effectively and readily employed for the treatment and prophylaxis of ZIKV infection in humans.

## Results

### CQ protects human neural progenitors from ZIKV infection

**Figure 1:**
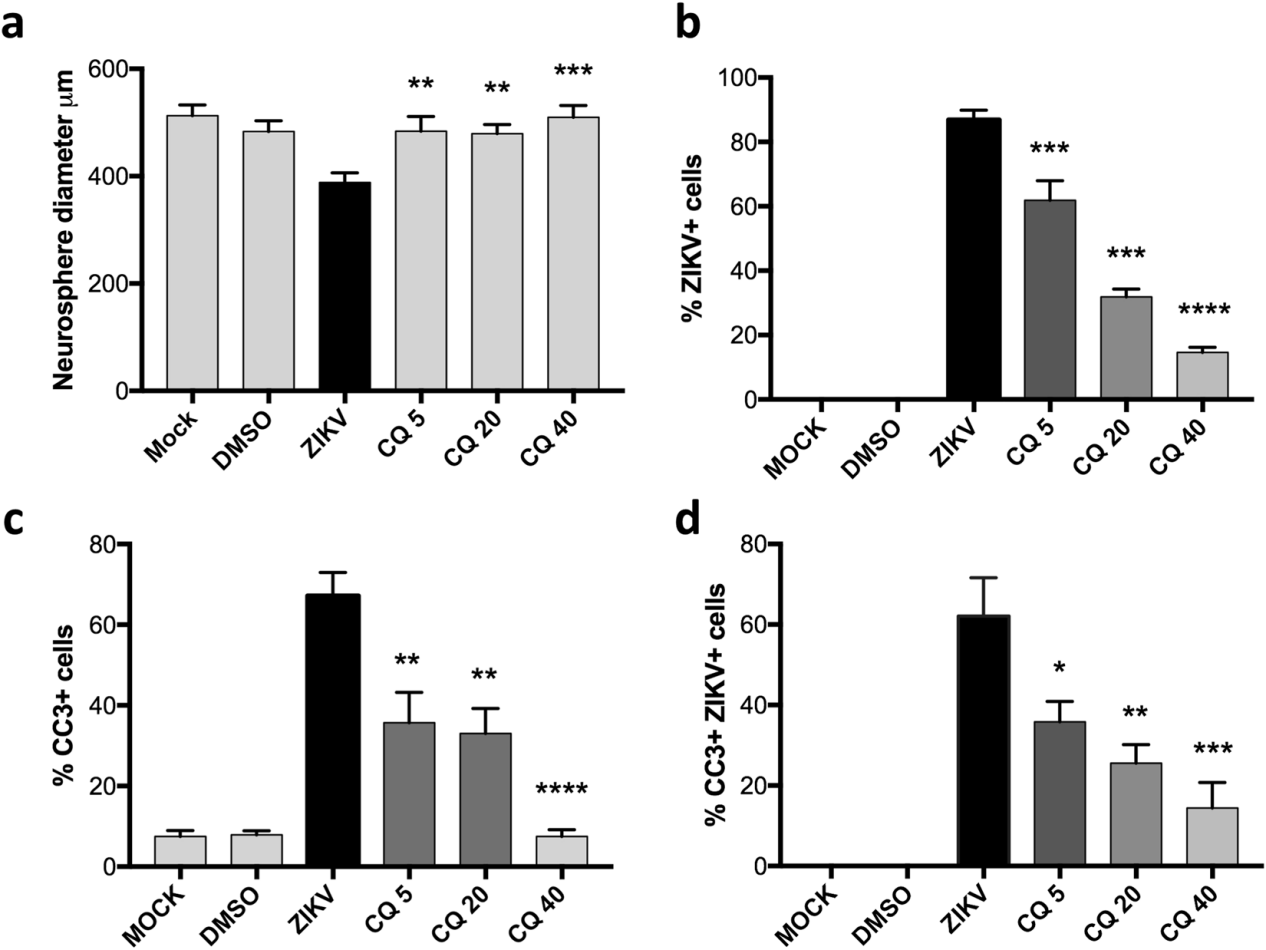
CQ inhibits ZIKV infection and apoptosis in human neurospheres. (**a**–**d**) Human iPSC-derived neurospheres were infected with ZIKV^BR^ (multiplicity of infection = 1) and immediately treated with CQ at 5 µM, 20 µM, or 40 µM. MOCK = uninfected neurospheres. DMSO = DMSO treated neurospheres. Quantification of (**a**) neurosphere size at 96 h post-infection, (**b**) ZIKV-positive cells, (**c**) cleaved caspase 3 (CC3)-positive cells, and **(d)** CC3 and ZIKV double-positive cells. Data are the mean ± SEM of triplicates. *p < 0.05, **p < 0.01, ***p < 0.001, ****p < 0.0001 compared with “ZIKV” one-way ANOVA with Dunnett’s multiple comparisons test.

Human foetal NPCs are the major target of ZIKV in the developing brain^5,15^. To examine the effect of CQ *in vitro*, we infected monolayer cultures of primary human foetal NPCs with ZIKV^BR^ (Brazilian strain ZKV2015) and cultured them in the absence or presence of up to 40 μM CQ. Consistent with work by others^12^, we found that CQ efficiently (90% inhibition at 6 µM) reduced ZIKV^BR^ infection of primary human foetal NPCs. To mimic ZIKV^BR^ infection in the context of the 3-dimensional architecture of the developing human brain, we examined neurospheres derived from human iPSCs (Fig. 1a). We found that CQ treatment reduced both the percentage of ZIKV^BR^-positive cells (Fig. 1b) and the level of apoptosis in the neurospheres with an IC_50_ of ~10 μM (Fig. 1c and d).

### CQ attenuates acute ZIKV-induced mortality in AG129 mice

To corroborate the *in vitro* findings, we first examined AG129 mice, which lack receptors for type I (α/β) and type II (γ) IFNs and have previously been used to model ZIKV infection^16,17^. To test the prophylactic effects of CQ, mice were administered 50 mg/kg/day CQ in drinking water for 2 days and then infected with ZIKV^BR^ (2 × 10^3^ PFU retro-orbitally). CQ treatment was continued at the same dose for 5 days and then at 5 mg/kg/day until the end of the experiment. Control mice received drinking water alone.

**Figure 2:**
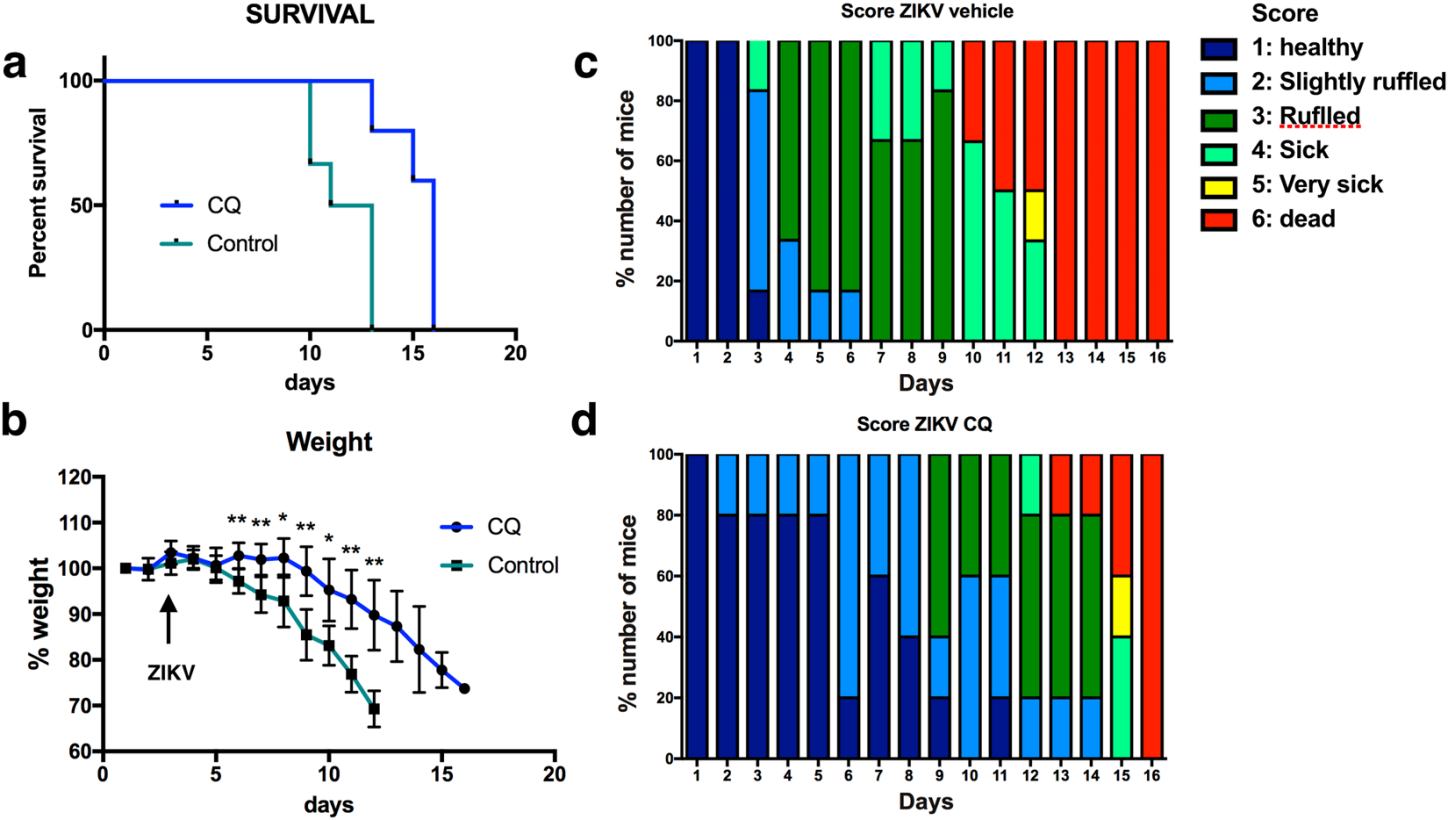
CQ attenuates ZIKV infection in AG129 mice. **(a)** Survival curves for control and CQ- treated mice (n = 6 and 5, respectively). Note that 60% of the CQ-treated mice were euthanized on day 15. *p* = 0.0075 by log-rank Mantel–Cox test. (**b)** Weight loss following ZIKV infection in control and CQ-treated mice. Data are the mean ± SEM. *p < 0.05, **p < 0.01 compared with controls by unpaired t-test with Welch’s correction. **(c**,**d)** Visual appearance health scores in ZIKV-infected control (**c**) and CQ-treated (**d**) mice. Note that by day 13, all control mice had died but 60% of CQ-treated mice were alive.

We observed that CQ extended the average lifespan of ZIKV-infected AG129 mice to 15 days (*p* < 0.01, log-rank Mantel–Cox test; Fig. 2a) and significantly attenuated ZIKV-induced weight loss (*p* < 0.01, unpaired t-test with Welch’s correction; Fig. 2b). Overall animal health was assessed using a modified 6-point scoring system^15^, which showed that CQ-treated mice remained in good health and survived for longer than the vehicle-treated mice (80% vs 0% of animals alive on day 13, respectively) (Fig. 2c and d). These results indicate that CQ attenuated disease severity in ZIKV-infected AG129 mice, which is considered the most severe model of ZIKV infection^16^.

### SJL mice support chronic infection with ZIKV

Mice deficient in IFN response genes, such as single knockout (*Ifnar1*) A129, double knockout (*Ifnar1*, *Ifnar2*) AG129, and triple knockout (*Irf3*, *Irf5*, *Irf7*) TKO mice^16^, succumb to ZIKV within a few days of infection making it difficult to investigate vertical transmission of ZIKV in such an aggressive disease model. Therefore, we explored the SJL mouse model, which we have previously used to study foetal transmission with high doses of ZIKV^4^.

**Figure 3:**
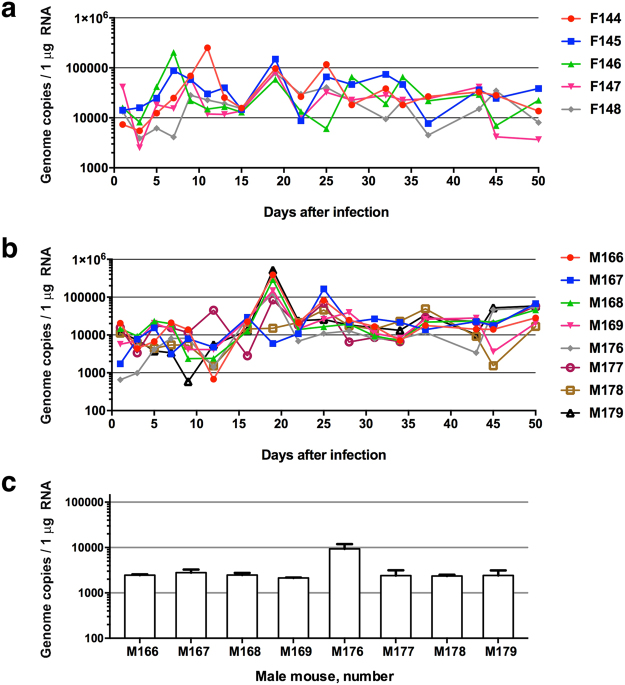
Mouse model of chronic ZIKV infection for testing therapeutic interventions. (**a)** Female (n = 5) and **(b)** male (n = 8) SJL mice were infected with 10^8^ PFU ZIKV^BR^ retro-orbitally. Blood samples were taken every 3 days for 50 days and analysed for viral RNA by qRT-PCR. **(c)** qRT-PCR analysis of viral RNA in the testes of male mice 3 months after ZIKV^BR^ infection. Mean ± SEM of 8 mice.

SJL males and females at 3 months of age were infected with ZIKV^BR^ (1 × 10^8^ PFU retro-orbitally), and circulating ZIKV RNA levels were analysed by qRT-PCR over the following 50 days. Our qRT-PCR assay is only 10-fold less sensitive than a laborious and time consuming plaque forming unit assay. Using qRT-PCR we could detect the levels of ZIKV as low as 10 plaque forming units per sample. In testing our samples, we did not record any ZIKV in the samples obtained from uninfected control mice. We found that the viral titres fluctuated over time in both males and females, ranging from 5 × 10^3^ to 4 × 10^5^ genome copies/µg total RNA. However, the mean titres were maintained between 10^4^ and 10^5^ genome copies/µg total RNA (Fig. 3a and b). Previous work has shown that ZIKV inoculation of wild-type C57BL/6 mice treated with a single dose of IFNAR1-blocking monoclonal antibody leads to infection of and damage to the testes^18^. We therefore investigated ZIKV titres in the testes of chronically infected SJL mice (3 months post-infection) and found readily detectable levels (10^3^–10^4^ ZIKV genome copies/1 µg testis RNA) (Fig. 3c). Collectively, these data indicate that SJL males and females support sustained ZIKV infection and display no signs of morbidity at 3 months post-infection. The mice therefore represent a physiologically relevant model for studying paternal and maternal vertical transmission of ZIKV^BR^.

### Vertical and horizontal transmission of ZIKV in SJL mice

**Figure 4:**
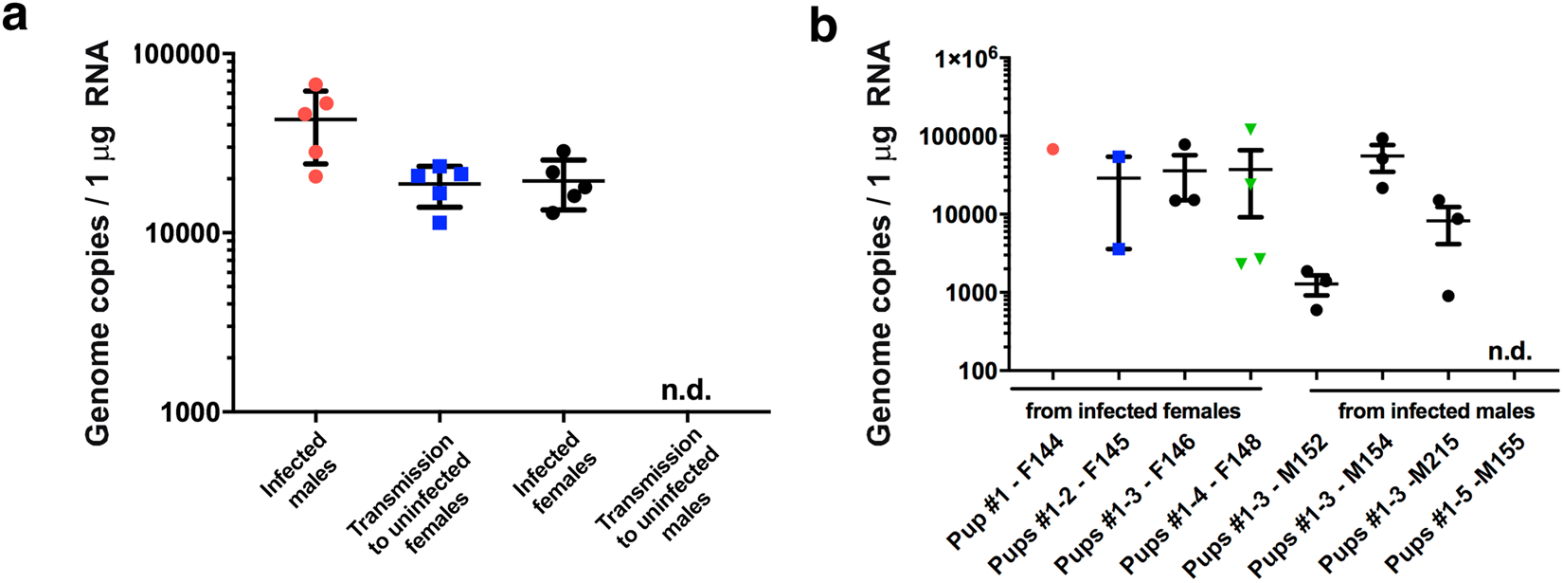
Horizontal and vertical sexual transmission of ZIKV in SJL mice. (**a**) Efficient sexual transmission of ZIKV^BR^ was observed from infected males to uninfected females, but not from infected females to uninfected males. SJL male and female mice (n = 5) were infected with ZIKV^BR^ (10^8^ PFU retro-orbitally) and co-housed for 14 days with uninfected females and males, respectively. Viral RNA was measured by qRT-PCR of serum samples obtained from the sires and dams on day 14. Each point represents one animal. Data are the mean ± SD. n.d.: not detected (**b)** Vertical transmission of ZIKV^BR^ to the offspring of infected sires and dams. RNA from the newborn heads was prepared on postnatal day 1 for qRT-PCR analysis of viral RNA. Each point represents one day 1 pup. Data are the mean ± SEM of the offspring of 4 infected dams/uninfected sires or 4 infected sires/uninfected dams as indicated.

We examined horizontal transmission by infecting 3-month-old SJL males and females with ZIKV^BR^ (10^8^ PFU retro-orbitally) and allowing them to mate with uninfected mice of the opposite sex. After 14 days, the uninfected males were separated and bled and circulating viral titres were measured by qRT-PCR. To avoid stress during pregnancy dams were allowed to deliver and then bled on the next day and circulating viral titres were measured by qRT-PCR. Interestingly, we observed efficient transmission of ZIKV from infected males to females but not vice versa (Fig. 4a). This is strikingly similar to the mode of horizontal transmission in humans, where female-to-male transmission is relatively rare^19–21^. Because we could not detect any ZIKV transmission from infected female mice to uninfected males, we concluded other routes of transmission such as via saliva or ocular secretions are insignificant.

Our previous study investigated foetal development in SJL females directly infected with a 4 × 10^10^ PFU/ml of ZIKV^BR^ on E12.5^4^. Here, we investigated whether the virus could be transmitted vertically from ZIKV-infected dams and sires to their offspring through the natural breeding process. To this end, 3-month-old female and male SJL mice were infected with ZIKV^BR^ (10^8^ PFU retro-orbitally) and immediately allowed to breed with uninfected mice of the opposite sex. After regular delivery, the 1-day-old pups were euthanized and tissue samples were analysed for viral RNA by qRT-PCR. We found that all dams efficiently transmitted ZIKV to their pups (Fig. 4b). Notably, transmission from the infected sires to their pups occurred in fewer animals was less efficient (Fig. 4b), possibly reflecting variable ZIKV titres in the semen and variations in the levels of sexual transmission of ZIKV from infected sires to dams. The molecular and cellular mechanisms of ZIKV infection during pregnancy are poorly understood^22^, and such knowledge is critical for the development of treatment to limit ZIKV infection during pregnancy. Our results thus demonstrate that SJL males and females can transmit ZIKV vertically through the natural mating process and thus represent a unique physiological mouse model for testing of drugs that could suppress vertical viral transmission.

### CQ suppresses vertical transmission of ZIKV

**Figure 5:**
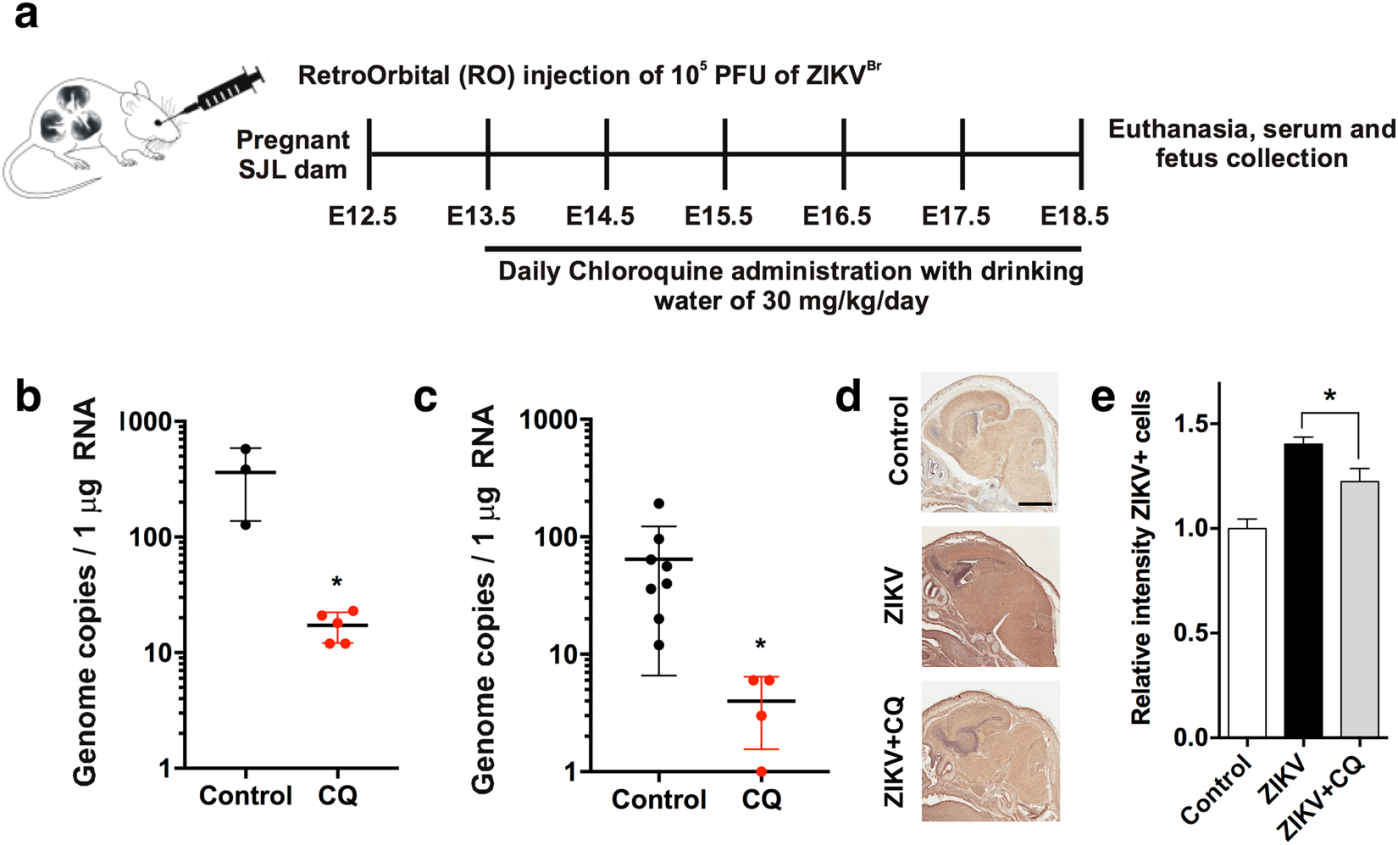
CQ represses ZIKV infection in SJL mice and reduces vertical transmission. (**a**) Schematic of the experimental design. SJL dams were infected with ZIKV (2 × 10^5^ PFU) on E12.5. On E13.5, they were randomly assigned to receive vehicle or CQ (30 mg/kg/day) in the drinking water. On E18.5, mice were euthanized for collection of blood and foetuses. (**b)** qRT-PCR of viral RNA. Each point represents one animal. Data are the mean ± SD of 3 (vehicle-treated) or 5 (CQ-treated) mice. *p < 0.05 by Student’s t-test. **(c)** qRT-PCR of viral RNA in foetal head extracts. Each point represents one foetus. Data are the mean ± SD of 8 foetuses pooled from 3 independent litters (control) or 5 foetuses pooled from 2 independent litters (CQ). *p < 0.05 by Student’s t-test. (**d)** Representative images of foetal brain sections from control, ZIKV-infected, and ZIKV-infected/CQ-treated mice on E18.5. Sections were stained with a primary antibody against Flavivirus Group Antigen (brown) and counterstained with Mayer’s hematoxylin (blue). Scale bar, 4 mm. **(e)** Quantification of ZIKV-infected cells in foetal brain sections from control, ZIKV-infected, and ZIKV-infected/CQ-treated mice. Data are the mean ± SEM of 6 sections per condition (3 embryos, 2 sections per embryo). *p < 0.05 compared with untreated ZIKV-infected mice by one-way ANOVA with Dunnett’s multiple comparisons test.

Next, we examined the effect of CQ treatment on vertical transmission of ZIKV in SJL mice using our previously published protocol^4^. Pregnant SJL mice (2–3 months of age) were infected with ZIKV^BR^ (2 × 10^5^ PFU retro-orbitally) on day E12.5. This dose is sufficient to cause a robust ZIKV infection in SJL mice. Infected dams were provided with CQ (30 mg/kg/day in drinking water) starting on day E13.5 and were euthanized on E18.5, at which point maternal blood and foetal brain samples were collected and analysed by qRT-PCR (Fig. 5a). This lower, 30 mg/kg, dosage of CQ was specifically use to protect pregnant mice from potential negative effects of the drug. We found that treatment with CQ reduced by ~20-fold the ZIKV titre in both maternal blood (Fig. 5b) and foetal brain (Fig. 5c). To confirm these results, whole embryos were immunostained with an anti-ZIKV envelope protein antibody. Consistent with the qRT-PCR data, this analysis revealed a significant reduction in the ZIKV immunostaining intensity in the foetuses of CQ-treated pregnant mice compared with the untreated mice (Fig. 5d and e).

## Discussion

CQ has been used worldwide for more than half a century for anti-malaria prophylaxis and therapy without evidence of foetal harm^23–26^. CQ can cross the placental barrier and would be expected to reach similar concentrations in maternal and foetal plasma^27^. The side effects of CQ have been thoroughly evaluated in a malaria prophylaxis study (400 mg/week), which found no increase in the incidence of birth defects^11^. High CQ concentrations (up to 500 mg/day) were administered to pregnant women with severe lupus or rheumatoid arthritis. Although a few instances of spontaneous abortion were observed (likely a consequence of the disease itself), all term deliveries resulted in normal healthy newborns^28^, suggesting that high doses of CQ do not interfere with foetal development in humans. The dosages of CQ we employed in our study were either comparable or significantly lower relative to the acceptable and widely used dosages in humans. Studies in rodent models have found that brain concentrations of hydroxychloroquine (CQ analogue) are 4–30 times higher than plasma concentrations^29^, suggesting that it has a favourable pharmacokinetic profile for inhibition of ZIKV infection in NPCs. In arthritis patients, plasma CQ concentrations reached 10 µM after daily administration of 5 mg/kg/day for a week^30^. Given that the half-life of CQ in humans is approximately 40 days^31^, people treated with 5 mg/kg/day CQ for 7 days will build up over 30 mg/kg of CQ, which is comparable to the regimen used in our animal studies. CQ treatment can be associated with retinopathy, but the reported threshold dose in humans, 5.1 mg/kg/day^30^, thus allowing sufficient accumulation of CQ (see above). Moreover, eye disease was not detected in a study of more than 900 rheumatoid arthritis patients treated with up to 4.0 mg/kg/day CQ for an average of 7 years^30^. Therefore, a level of CQ sufficient to protect SJL mice from ZIKV could be safely build up in a human body in relatively short, 7 days, time period and then maintained for many weeks or months with a minimal intake of CQ. The pharmacokinetics of CQ thus make it an excellent candidate for prophylaxis in individuals at high risk of ZIKV infection (e.g., residents or visitors in ZIKV endemic areas). Our results demonstrate that CQ effectively reduces ZIKV infection in primary human foetal NPCs and in two mouse models, and that CQ at doses comparable to or less than those broadly used in humans can markedly reduce maternal and foetal infection.

ZIKV infects cells through receptor-mediated endocytosis and membrane fusion within acidic endosomes^32^. CQ is thought to affect acidification of the endosomes and thus obstructs fusion of the flaviviral envelope protein with the endosomal membrane^32^. Cellular proteases, including furin, are essential for cleavage of the flaviviral prM during viral egress^33^. This transition is pH-dependent, and alterations in the intracellular pH may result in the release of less infectious virions^34^. Clearly, additional studies are required to determine the precise pharmacological mechanism by which CQ counters ZIKV activity.

Several approved and experimental drugs have been identified as potential anti-ZIKV agents, including daptomycin, 25-hydroxycholesterol, emricasan, niclosamide, azithromycin and sofosbuvir, and 7-deaza-2′-C-methyladenosine (inhibitors of the viral RNA polymerase)^35–41^. Thus, similar with CQ, 7-deaza-2′-C-methyladenosine (50 mg/kg/day) delayed ZIKV-induced morbidity and mortality in AG129 mice^41^. Further research and clinical trials will be needed to determine the optimal individual or combination drugs, for example, such as CQ and sofosbuvir or 7-deaza-2′-C-methyladenosine, for ZIKV therapy and prophylaxis. However, the long-term safety of CQ, even in pregnant women, as well as its low cost and worldwide availability, support its use alone or in combination (e.g., with sofosbuvir^37,42^) for the treatment and prophylaxis of ZIKV.

## Methods

### 3-Dimensional human neurosphere assay

Control NPCs were derived from human iPSCs (WT83 c9 and c6), as previously described^4^. All iPSC lines were negative for mycoplasma contamination. Briefly, high-passage iPSC/hESC colonies on feeder-free plates were grown to 80% confluency. The medium was changed to N2 (DMEM/F12 medium containing 1 × N2 supplement [Invitrogen], 1 μM dorsomorphin [Tocris], and 1 μM SB431542 [Stemgent]) for 48 h. Colonies were then detached from the plate and cultured in suspension as embryoid bodies for 5 days at 90 rpm in N2 medium. Embryoid bodies were plated on Matrigel (Corning) in NGF medium (DMEM/F12 medium supplemented with 0.5 × N2, 0.5 × B27 supplement [Gibco], 20 ng/ml fibroblast growth factor, and 1% penicillin/streptomycin) for 1 week. Rosettes were manually picked, dissociated, and added to plates double-coated with polyornithine (10 μg/mL, Sigma-Aldrich) and laminin (2.5 μg/mL, Gibco) in NGF medium. To generate neurospheres, NPCs were scraped from the plates and continuously shaken for 2–5 days at 90 rpm in NGF medium. Neurospheres were then treated with NG medium supplemented with 10 μM ROCK inhibitor (Y-27632, Calbiochem) for 48 h. Neurospheres were maintained in NG medium for 4 weeks to allow neuronal maturation.

### Neurosphere infection and treatment

Neurospheres were dissociated with Accutase (Thermo Fisher) and counted. For each assay, ~20 neurospheres/condition were treated as follows: uninfected (MOCK on figures), uninfected and treated with DMSO, infected with ZIKV^BR^ at a multiplicity of infection of 1, or ZIKV infected and treated with CQ at 5, 20, or 40 µM. CQ was added during viral adsorption (1 h at 37 °C). Medium containing the appropriate concentration of CQ was changed after 2 days. At 96 h post-infection, neurospheres were transferred to polyornithine/laminin double-coated plates and maintained for 1 week to initiate neuronal maturation. Medium supplemented with CQ was changed every 2–3 days.

### Immunofluorescence staining

Neurospheres were stained with anti-MAP2 (ab5392, Abcam), anti-Flavivirus Group Antigen (MAB10215, Millipore), anti-cleaved caspase 3 (9661, Cell Signaling), and DAPI. At least 3–6 images were acquired for each condition. DAPI was used to normalise cell numbers. Quantification of ZIKV^BR^-positive cells was based on colocalisation of Flavivirus, MAP2, and DAPI staining.

### Histology and immunohistochemistry

Mouse embryos obtained on E18.5 were fixed for 48 h in 4% formaldehyde in phosphate-buffered saline (PBS), transferred to sucrose, and embedded in paraffin. Serial sections (5 μm) were cut along the sagittal axis of the embryo. Slides were deparaffinised and rehydrated using xylene and graded ethanol. Antigen retrieval was performed in a pressure cooker at 7.5 psi in 0.1 M Tris-HCl buffer (pH 9.0) for 15 min. Slides were rinsed with water 6 times at room temperature and washed for 5 min in PBS. Endogenous peroxidase activity was quenched by incubation in 3% hydrogen peroxide in PBS for 30 min at room temperature. Slides were incubated for 16–18 h at 4 °C with primary anti-Flavivirus Group Antigen (Millipore, #MAB10216) diluted 1:250 in Dako Antibody Diluent with Background Reducing Components (Agilent, #S3022). After rinsing in PBS 3 times for 5 min each, the slides were incubated with a horseradish peroxidase-conjugated goat anti-mouse secondary antibody (Abcam, #ab2891) for 30 min at room temperature. Slides were washed again in PBS, incubated for 3 min with DAB complex (ImmPACT DAB Peroxidase Substrate, Vector Laboratories, #SK-4105), and washed 3 times each in PBS and water. Finally, slides were counterstained in Mayer’s hematoxylin, mounted, visualised, and recorded using an Aperio automated system (Leica). The images were analysed using ImageScope (Leica).

### Animal studies

All animal procedures were performed in accordance with the PHS Policy on Humane Care and Use of Laboratory Animals and with the approval of the Sanford Burnham Prebys Medical Discovery Institute IACUC Committee, protocol AUF#16-049.

### ZIKV infection in AG129 mice

Mice received CQ (50 mg/kg in drinking water) or drinking water alone for 2 days prior to infection. ZIKV^BR^ (2 × 10^3^ PFU) was injected retro-orbitally, and CQ treatment was continued at 50 mg/kg in drinking water for 5 days and then at 5 mg/kg/day for the remainder of the experiment. A 6-point visual health scoring system was modified from the 7-point scale previously published by Tang *et al*.^15^. According to institutional IACUC requirements, moribund animals were euthanized and not scored. Animals were considered moribund if they could not turn themselves upright when placed on their sides.

### Model of chronic ZIKV infection in SJL mice

Two groups of 3-month-old SJL mice (8 males and 5 females) were infected with ZIKV^BR^ (1 × 10^8^ PFU retro-orbitally), and blood samples were collected every third day for 50 days. Males were euthanized at 3 months post-infection to obtain testis samples. Viral RNA titres were measured by qRT-PCR analysis of serum and testis RNA.

### ZIKV infection in SJL mice

Two- to three-month-old pregnant SJL mice (Jackson Laboratories, Bar Harbor, ME, USA) were infected on E12.5 with ZIKV^BR^ (2 × 10^5^ PFU retro-orbitally). CQ (30 mg/kg in drinking water) was provided for 5 days between E13.5 and E18.5. We regularly monitored and tracked water consumption in our experimental and control groups. We did not observe any difference in water consumption between the groups. On E18.5, maternal blood samples were collected and the animals were euthanized to obtain the foetal tissues. In order to prevent contamination of foetal samples from maternal blood the pregnant mice were transcardially perfused (day 18.5) with 10 ml PBS to fully remove the blood from animals.

### Horizontal and vertical transmission of ZIKV in SJL mice

Three-month-old SJL male and female mice (Jackson Laboratories, Bar Harbor, ME, USA) were infected with ZIKV^BR^ (10^8^ PFU retro-orbitally) and then allowed to breed with uninfected females or males, respectively. After 14 days, blood samples were collected from males. On day 1 post-delivery, blood samples were collected from the mothers and the pups were euthanized to obtain brain tissues. Viral RNA levels in the blood and brain tissues were measured by qRT-PCR.

### Real-time qPCR quantification of ZIKV

Total RNA was extracted from samples of blood (0.1 ml), foetal br–in, or pups using a NucleoSpin RNA Kit (Macherey-Nagel). RNA concentrations were measured using a NanoDrop spectrophotometer (NanoDrop Technologies) and the samples were kept at −80 °C until use. ZIKV-specific primers were ZIKV-835 5′-TTGGTCATGATACTGCTGATTGC-3′ and ZIKV-911c 5′-CCTTCCACAAAGTCCCTATTGC-3′, as described previously^4,43^. Real-time PCR was performed using QuantiTect Reverse Transcription Kit (QIAGEN), SYBR Green I Master Mix, and a LightCycler 480 II instrument (Roche) using the following conditions: initiation at 95 °C for 10 min followed by 50 cycles of 95 °C for 15 s, 60 °C for 30 s, and 72 °C for 30 s. Data were analysed using LightCycler 480 Software 1.5.0 (Roche). Assay sensitivity was determined using samples with known ZIKV concentrations. GraphPad Prism was used as the fitting software.
